# Evaluation of a Commercial Multiplex PCR Assay for Detection of Pathogen DNA in Blood from Patients with Suspected Sepsis

**DOI:** 10.1371/journal.pone.0167883

**Published:** 2016-12-20

**Authors:** Ingrid Ziegler, Anna Fagerström, Kristoffer Strålin, Paula Mölling

**Affiliations:** 1 Department of Infectious Diseases, Örebro University Hospital, Örebro, Sweden; 2 School of Health and Medical Sciences, Örebro University, Örebro, Sweden; 3 Department of Laboratory Medicine, Örebro University Hospital, Örebro, Sweden; 4 Department of Infectious Diseases, Karolinska University Hospital, Stockholm, Sweden; 5 Department of Medicine Huddinge, Karolinska Institutet, Stockholm, Sweden; US Army Medical Research Institute of Infectious Diseases, UNITED STATES

## Abstract

The Magicplex Sepsis Real-time Test (MST) is a commercial multiplex PCR that can detect more than 90 different pathogens in blood, with an analysis time of six hours. The aim of the present study was to evaluate this method for the detection of bloodstream infection (BSI). An EDTA whole blood sample for MST was collected together with blood cultures (BC) from patients with suspected sepsis at the Emergency Department of a university hospital. Among 696 study patients, 322 (46%) patients were positive with at least one method; 128 (18%) were BC positive and 268 (38%) were MST positive. Considering BC to be the gold standard, MST had an overall sensitivity of 47%, specificity of 66%, positive predictive value (PPV) of 23%, and a negative predictive value of 87%. Among the MST positive samples with a negative BC, *coagulase-negative staphylococci* (*CoNS*) and species that rarely cause community-acquired BSI were frequently noted. However, the quantification cycle (Cq) values of the MST+/BC- results were often high. We thus hypothesized that the performance of the MST test could be improved if the Cq cut-off level was adjusted downwards. With a lower Cq cut-off value, i.e. 6.0 for *Staphylococcus* species and 9.0 for all other species, the number of MST positive cases decreased to 83 (12%) and the overall sensitivity decreased to 38%. However, the PPV increased to 59% and the specificity increased to 96%, as many MST positive results for *CoNS* and bacteria that rarely cause community-acquired BSI turned MST negative. In conclusion, our study shows that with a lower Cq cut-off value, the MST will detect less contaminants and findings with unclear relevance, but to the cost of a lower sensitivity. Consequently, we consider that a positive MST results with a Cq value above the adjusted cut-off should be interpreted with caution, as the result might be clinically irrelevant. In a correspondent way, quantitative results could probably be useful in the interpretation of positive results from other molecular assays for the detection of BSI.

## Introduction

Sepsis is a major cause of morbidity and mortality [1, 2]. Early, accurate administration of antibiotics is the most important intervention for successful outcome in this serious condition [3, 4]. In international guidelines [5] it is recommended that effective antibiotic treatment should be given as soon as sepsis is suspected, preferably within one hour. Broad-spectrum antibiotics are often recommended as empiric treatment. However, with the emerging problem of antibiotic resistance, it is essential to administer narrow-spectrum antibiotics, more specifically targeting the causative pathogen, as soon as possible.

Blood culture (BC) is the current ‘gold standard’ for diagnosing bloodstream infections (BSI), but the method has several limitations. Sensitivity decreases greatly when antibiotics have been given prior to culture [6, 7], and slow-growing, fastidious organisms can be difficult to detect by BC [8]. It has been shown that a positive BC is found in around only half of patients with sepsis and septic shock [9, 10]. Furthermore, it takes about 24–72 hours before the pathogen is identified and information about antibiotic susceptibility is available [8]. Consequently, changes in empirical antibiotic therapy are more frequently guided by clinical response than culture results [11]. With new techniques, such as matrix-assisted laser desorption ionization-time of flight (MALDI-TOF) or the FilmArray Blood Culture Identification Panel, the time to identification can be significantly shortened, and it has recently been described that this reduction can lead to a significantly shorter time to accurately targeted antibiotic treatment [12, 13].

For optimal empirically selected antibiotic therapy in sepsis, techniques on whole blood for the direct and rapid detection of causative agents are essential. Molecular methods based on polymerase chain reaction (PCR) technology have been developed for this purpose. By using PCR methods on blood samples, it is possible to detect pathogen DNA in blood within hours.

There are a few commercial tests available for the direct molecular identification of pathogens in blood samples [14–16]. Out of these tests, only the Light Cycler SeptiFast Test (Roche Diagnostics) has, to date, been well investigated in clinical studies. In a meta-analysis upon the SeptiFast test, Dark et al [17] concluded that the method appeared to have higher specificity than sensitivity, with the reservation of deficiencies in study quality.

More recently, the promising IRIDICA PCR/electrospray ionization—mass spectrometry (PCR/ESI-MS) technology (Abbot), has been launched, but so far this method has only been evaluated in a few study-cohorts [18, 19].

The Magicplex Sepsis Real-time PCR (SeeGene, Seoul, Korea) is one of the most recent commercial devices for this purpose. This multiplex PCR is able to detect 73 gram-positive and twelve gram-negative bacteria, three drug resistance markers (*mecA*, *vanA* and *vanB*) and six fungi. So far, clinical validation has been sparse, with only a few studies published on the Magicplex Sepsis Real-time test (MST) [20–22]. All these studies, including one study with 375 patients, conducted by Ljungström et al [22], one with 267 patients, conducted by Carrara et al [20] and one with 125 patients, conducted by Loonen et al [21], found many MST positive cases that were not supported by other traditional microbiological tests, and where the clinical picture was doubtful. Based on these findings, we hypothesized that the MST assay may detect low levels of bacterial DNA without clinical relevance, and that the method could possibly be more useful after adjustment downwards of the detection cut-off value.

The aim of the present study was to compare BC with the MST assay using different quantification cycle (Cq) cut-offs in a large study cohort including unselected patients with suspected sepsis. To evaluate the accuracy of positive PCR results without BC-support, such samples were analyzed with another, species-specific PCR method.

## Materials and Methods

### Patients

This study was performed at the Emergency Department of Örebro University Hospital, Sweden, between February 2011 and June 2012. During the study period, an EDTA whole blood sample was collected from the same venous puncture as BC samples in consecutive patients subjected to blood culturing. The EDTA blood samples were stored in a Biobank. The Regional Medical Ethics Review Board of Uppsala, Sweden, approved the use of the blood samples for this study, provided that they were made anonymous, and that no patient data were available other than information on gender, age and BC result. No informed consent was thus required from the patients.

### Blood culture

Two pairs of BC samples, i.e. four BC bottles, were collected per patient, according to routine practice. For each BC, a volume of 8–10 mL of venous blood was inoculated in one Bactec Aerobic/F bottle and the same volume in one Bactec Plus Anaerobic /F bottle. The BC bottles were incubated in a Bactec blood culturing system (Becton Dickinson and Company, Sparks, MD, USA) for up to seven days.

### Magicplex sepsis real-time Test (MST)

Within three days after sample collection, bacterial DNA was extracted from 1 mL EDTA whole blood, using the Blood Pathogen Kit (Molzym, Bremen, Germany) on an Arrow instrument (Diasorin, Solna, Sweden), following the manufacturer’s instructions, and frozen at -70°C pending the PCR run. All runs included a positive and a negative control.

Pathogen detection was performed using the Magicplex Sepsis Real-time Test. According to the manufacturer, the MST has a limit of detection (LOD) of 30 colony-forming units (CFU)/mL. The LOD was tested using blood samples spiked with *Staphylococcus aureus*, *Streptococcus pneumoniae*, *Pseudomonas aeruginosa* and *Candida albicans* respectively, at known concentrations. All pathogens with concentrations ≥ 30 CFU/mL were detected, but below 30 CFU/mL the detection rate declined.

The MST process is summarized in Table 1.

**Table 1:** Process of the Magicplex Sepsis Real-time Test.

**Step 1: Amplification on PCR, 2.5 h**					
**Amplicon bank 1**			**Amplicon bank 2**		
**Gram-positive bacteria/drug resistance:**			**Gram-negative bacteria/fungi:**		
73 Gram-positive bacteria, three drug resistance markers			12 gram-negative bacteria/six fungi		
**Step 2: Screening on Real-time PCR, 30 min**					
**Gram-positive bacteria screening**		**Drug resistance screening**		**Gram-negative bacteria/ Fungi Screening**	
*Streptococcus* spp *Enterococcus* spp *Staphylococcus* spp		vanA, vanB, mecA		Gram-negative bacteria-A Gram-negative bacteria-B	
**Step 3: Identification on Real-time PCR, 30 min**					
**ID 1. *Streptococcus* spp.**	**ID 2. *Enterococcus* spp.**	**ID 3. *Staphylococcus* spp.**	**ID 4–5. Gram-negative bacteria-A**	**ID 6–7. Gram-negative bacteria-B**	***ID 8–9*. *Fungi***
*Streptococcus agalactiae* *Streptococcus pyogenes* *Streptococcus pneumoniae*	*Enterococcus faecalis* *Enterococcus gallinarum* *Enterococcus faecium*	*Staphylococcus epidermidis* *Staphylococcus haemolyticus* *Staphylococcus aureus*	*Pseudomonas aeruginosa* *Acinetobacter baumannii* *Stenotrophom-onas maltophilia* *Serratia marcescens* *Bacteroides fragilis* *Salmonella typhi*	*Klebsiella pneumoniae* *Klebsiella oxytoca* *Proteus mirabilis* *Escherichia coli* *Enterobacter cloacae* *Enterobacter aerogenes*	*Candida albicans* *Candida tropicalis* *Candida parapsilosis* *Candida glabrata* *Candida enusei* *Aspergillus fumigatus*

Briefly, MST is a three-step procedure with an initial PCR amplification of target nucleic acids using 20 μL of extracted DNA in two different reactions, one for Gram-positive bacteria/drug resistance genes (GPB/DR) and one for Gram-negative bacteria/Fungi (GNB/Fungi). In the second step, screening for detection of more than 90 different pathogens at the genus level and resistance markers is performed using real-time PCR. This is done in three different reactions where 2 μL of the GPB/DR amplicon are used for screening of GPB /DR, and 2 μL of the GNB/Fungi amplicon are used for screening of GNB/Fungi. Species identification (without additional amplification) is accomplished in the third step where 2 μL of the initial amplicons are used with different Magicplex Sepsis ID (1–9) Real-time Detection kits, depending on the results of the previous screening PCR. Twenty-seven pathogens can be identified at the species level.

The amplification was performed on a GeneAmp 9700 (Applied Biosystems, Foster City, CA, USA) and all real-time PCR analyses were performed on the CFX96 Real-time PCR System (Bio-Rad Laboratories, Hercules, CA, USA).

### Quantification cycle, (Cq)

During PCR, the amount of PCR product is measured at each cycle and reported in fluorescence units. The more target-DNA present in a sample, the quicker a significant PCR product is generated. A sample is positive if the amount of fluorescence produced rises above a defined threshold level. This point is called the quantification cycle (Cq). Thus, the more target-DNA present in a sample, the lower the Cq value will be. According to the MST manufacturer, a Cq of <10 cycles is needed for positivity for *Staphylococcus* species, and a Cq of <15 cycles is needed for positivity for all other pathogens.

With the MST, a Cq value is generated both in the second (screening) step and in the third (identification) step. In the present study, we included Cq values from the identification step only, as they are specific for the species identified. For example, a sample containing DNA from both *Staphylococcus aureus* and *Coagulase negative staphylococci* (*CoNS*) will give two separate Cq values in the identification step, but just one in the screening step (*Staphylococcus* species).

### In-house real-time PCR

BC negative samples, with positive MST results for *S*. *aureus*, *Escherichia coli* or *S*. *pneumoniae*, were further analyzed using species-specific in-house real-time PCR protocols with the same DNA extractions as for MST. The *S*. *aureus* and *E*. *coli* PCR: s were performed on Rotorgene Q (Qiagen, Hilden, Germany) in a volume of 20 μL including 5μL template DNA.

The *S*. *aureus* PCR was run as described previously [23], however, adopted on a Rotorgene Q (Qiagen) using 0.3μM of each *nuc* primer. The *E*. *coli* PCR mix contained 1× Rotor-Gene Probe PCR Master Mix (Qiagen), 0.5 μM respective 0.3 μM of the primers *uidA* F and *uidA* R and 0.3μM of a probe *uidA* P2 [24]. The PCR was run at 95°C for 3 minutes followed by 40 cycles of 95°C for 3 seconds and 60°C for 10 seconds.

The PCR for *S*. *pneumoniae* (*lytA*) was performed on Lightcycler 2.0 (Roche Diagnostics, Mannheim, Germany) using a previously described protocol [25].

### Statistics

Medians and ranges were used for descriptive statistics of continuous variables, and percentages for categorical variables. Comparison of groups was performed using the Mann–Whitney U-test for continuous variables and the Chi-square or Fischer’s exact test for categorical variables. A p-value of < 0.05 was regarded as significant. Receiver-operating characteristic (ROC) curves were constructed to illustrate various cut-off levels. Area-under-curve values (AUC) were reported with 95% confidence interval (CI). Sensitivities, specificities and predictive values were calculated from cross-tabulations. The IBM SPSS Statistics, Version 21, New York, USA, was used for calculations.

## Results

The MST test was run on 703 blood samples from patients with suspected sepsis. Seven samples turned out to be invalid and were excluded. Consequently, samples from 696 patients were included in the study. Ten samples were MST positive only in the screening step (six positive for *Staphylococcus* species, two positive for *Streptococcus* species and two positive for the *mecA*-gene). However, in the present study, these samples were considered to be negative, as we required complete pathogen detection in the third step for a positive MST result.

**Fig 1:**
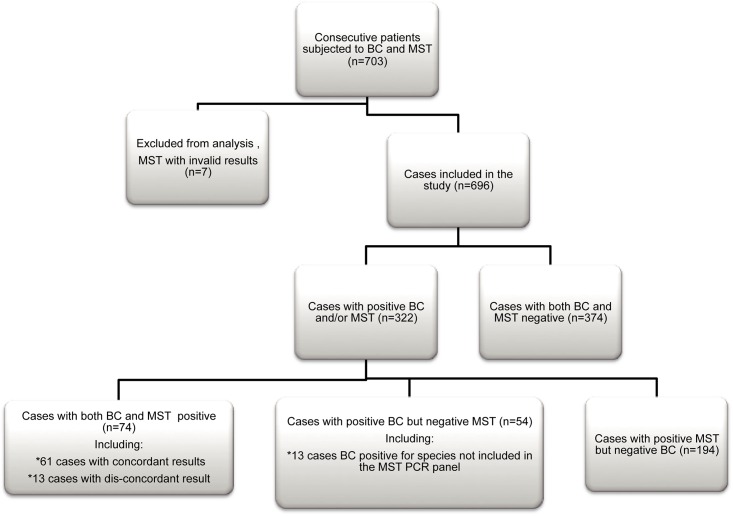
Flow-chart of study inclusion and overall results.

In Fig 1, the inclusion process and overall results are illustrated in a flow-chart. Table 2 shows the distributions of age and gender in the study population.

**Table 2:** Age, gender, and rate of positive blood culture (BC) and positive Magicplex Sepsis Test (MST) in the study population.

Factor	All study subjects (n = 696)	Subjects with positive BC (n = 128)	Subjects with positive MST (n = 268)	Subjects with positive MST with lower Ct cut-off value (n = 83)
Median age (range) in years	67(18–97)	74 (19–95)	68 (18–95)	73 (18–95)
Female sex, n (%)	294 (42)	56 (44)	118 (44)	34 (45)
BC positive, n (%)	128 (17)	all	61 (23)	49 (59)
MST positive, n (%)	268 (39)	61 (48)	all	all
MST positive with lower cut-off, n (%)	83 (12)	49 (38)	83 (31)	all

Three hundred and twenty-two (46%) samples were positive with at least one method; 128 (18%) were positive in the BC and 268 (39%) were positive in the MST. In 61 cases, both methods were positive for the same pathogen. Based on negative and positive results, agreement between BC and MST was noted in 435 (63%) cases. Seventy-eight samples were MST positive for more than one pathogen; three were positive for 4 pathogens, ten for 3 pathogens and sixty-five for 2 pathogens. In total, this resulted in 362 pathogens detected by MST. Nine samples were BC positive for two bacteria, and one sample was BC positive for 3 different pathogens.

MST+/BC+ cases had growth in all BC bottles more often than MST-/BC+ cases; 61% (37/61) versus 22% (15/67), p< 0.001. Regarding *CoNS*, growth in all BC bottles was noted in 22% (2/9) of MST+/BC+ cases and in 8% (2/25) of MST-/BC+ cases, p<0.001. The two cases with MST+/BC+ results for *CoNS* had MST Cq values of 4.3 and 6.3.

### Performance of MST compared to blood culture

Table 3 shows all MST and/or BC positive analyses for different species.

**Table 3:** Positive results for MagicPlex Sepsis Test (MST) and/or blood culture (BC), for different species.

Pathogen	According to original test result			With lower cut-off^a^		
	MST+/BC+	MST+/BC-	MST/BC+	MST+/BC+	MST+/BC-	MST-/BC+
*Staphylococcus aureus*	14	35	5	12	8	7
*Streptococcus pneumoniae*	6	15	7	5	2	8
*Streptococcus pyogenes*	2	0	1	2	0	1
*Streptococcus agalactiae*	1	25	0	1	3	0
*Streptococcus* spp	1	0	5	1	0	5
*Enterococcus faecalis*	4	7	3	3	3	4
*Escherichia coli*	20	30	17	16	6	21
*Klebsiella pneumoniae*	4	4	1	3	2	2
*Klebsiella oxytoca*	0	1	2	0	1	2
*Enterobacter cloacae*	3	5	0	3	1	0
*Proteus mirabilis*	0	0	1	0	0	1
*Pseudomonas aeruginosa*	0	4	3	0	0	3
*Acinetobacter baumannii*	0	11	0	0	1	0
*Stenotrophomonas maltophilia*	0	24	0	0	0	0
*Serratia marcescens*	0	1	0	0	0	0
*Bacteroides fragilis*	3	3	0	3	0	0
*Coagulase negative staphylococci*	9	129	16	3	10	22
*Candida species*	0	2	0	0	0	0
*Micrococcus species* ^b^	0	0	2	0	0	2
*Citrobacter species* ^b^	0	0	1	0	0	1
*Enterobacter sakazakii* ^b^	0	0	1	0	0	1
*Bacillus cereus* ^b^	0	0	1	0	0	1
*Actinobaculum schaalii* ^b^	0	0	1	0	0	1
*Propionebacterium acnes* ^b^	0	0	2	0	0	2
*Hemophilus influenzae* ^b^	0	0	2	0	0	2
*Salmonella species* ^b^	0	0	2	0	0	2
*Clostridium perfringens* ^b^	0	0	1	0	0	1
Total	66	296	74	51	37	89

^a^Ct cut-off 6.0 for *Staphylococcus* species and 9.0 for all other species.

^b^ Not included in the MST PCR panel

Considering BC as the gold standard, the MST test showed an overall sensitivity of 48% (61/128), a specificity of 66% (360/565), positive predictive value (PPV) of 23% (61/266), and negative predictive value (NPV) of 87% (374/428). For all individual pathogens in the study, the MST showed specificities of ≥95% and NPV of ≥97%. The sensitivities and PPV were 74% (14/19) and 29% (14/49) for *S*. *aureus*, 54% (20/37) and 40% (20/50) for *E*. *coli*, and 46% (6/13) and 29% (6/21) for *S*. *pneumoniae*, respectively.

As noted, 13 samples were BC positive for species not included in the MST test menu, i.e *Salmonella species*, *Haemophilus influenzae*, *Propionibacterium acnes*, *Micrococcus species*, *Citrobacter species*, *Bacillus cereus*, *Clostridium perfringens*, *Enterobacter sakazakii*, *Citrobacter species*, and *Actinobaculum schaalii*.

### Blood culture positivity in relation to Cq values

**Fig 2:**
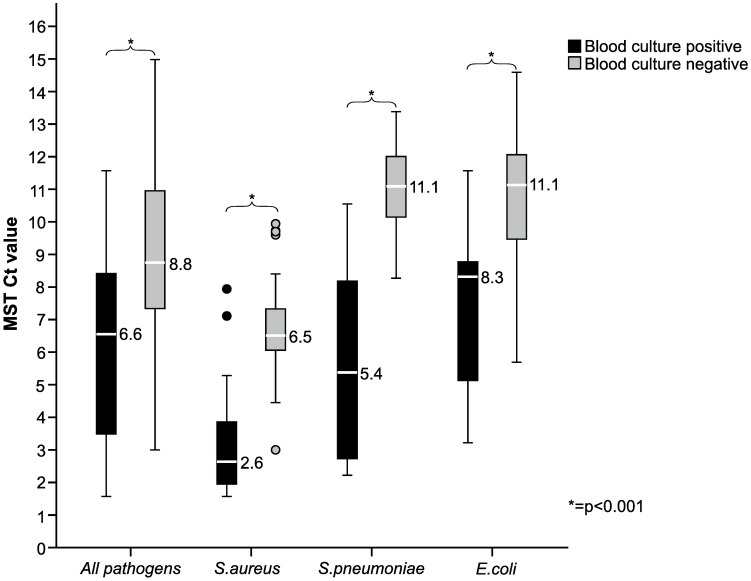
Cq values of specimens positive with the Magicplex Sepsis test (MST). Comparison of median Cq values between blood culture negative and blood culture positive samples among all MST positive samples, and samples with MST positive for *Staphylococcus aureus*, *Streptococcus pneumoniae* and *Escherichia coli*.

Among MST positive cases, those with concordant positive BC had significantly lower MST Cq values than cases with a negative BC (Fig 2). This pattern was also noted for the three largest pathogen groups, i.e. *E*. *coli*, *S*. *aureus*, and *S*. *pneumoniae*.

Based on these findings, we evaluated the performance of MST at different cut-off values.

ROC-curves were generated for MST detection of BSI with *S*. *aureus*, *E*. *coli* and *S*. *pneumoniae* at different MST Cq values. The AUC:s for MST detection were 0.860 for *S*. *aureus* BSI, 0.754 for *E*. *coli* BSI, and 0.723 for *S*. *pneumoniae* BSI.

Table 4 shows MST sensitivities, specificities and predictive values for BSI detection of *S*. *aureus*, *E*. *coli*, and *S*. *pneumoniae*, at different cut-off values.

**Table 4:** Sensitivities, specificities and predictive values of the Magicplex Sepsis Test (MST) at different cut-off Cq values for blood culture (BC) positivity, for the three most commonly detected sepsis-causing pathogens.

Species, cut-off Cq value	Sensitivity ^a^	Specificity^b^	Negative predictive value^c^	Positive predictive value ^d^	Expanded positive predicitive value^e^
***Staphylococcus aureus***					
**Any Cq value**	74(14/19)	95(642/677)	99(642/647)	29(14/49)	33(16/49)
**< 10**	74 (14/19)	95(642/677)	99(642/647)	29(14/49)	33(16/49)
**< 9**	74 (14/19)	95(646/677)	99(646/651)	31(14/45)	33(16/49)
**< 8**	74 (14/19)	96(647/677)	99(659/654)	33(14/42)	36(15/42)
**< 6**	63 (12/19)	99(669/677)	99(659/676)	60(12/20)	65(13/20)
**< 5**	58 (11/19)	99.7(675/677)	99(675/683)	85(11/13)	92(12/13)
***Escherichia coli***					
**Any Cq value**	54(20/37)	95(629/659)	97(615/646)	40(20/50)	46(23/50)
**< 10**	49(18/37)	98(649/659)	97(649/670)	64(18/28)	75(21/28)
**< 9**	43(16/37)	99(653/659)	97(653/674)	73(16/22)	86(19/22)
**< 8**	24(9/37)	99.7(657/659)	96(657/685)	82(9/11)	91(10/11)
**< 6**	16(6/37)	99.8(659/659)	95(658/689)	86(6/7)	100(7/7)
***Streptococcus pneumoniae***					
**Any Cq value**	46(6/13)	98(668/683)	99(668/675)	29(6/21)	33(7/21)
**< 10**	39(5/13)	99.7(681/683)	99(681/689)	71(5/7)	86(6/7)
**< 9**	39(5/13)	99.7(681/683)	99(681/689)	71(5/7)	86(6/7)
**< 8**	23(3/13)	100(683/683)	99(683/693)	100(3/3)	100(3/3)

^a^Reported as % (No. with positive MST and positive BC/Total no. with positive BC)

^b^Reported as %(No. with negative MST and negative BC/Total no. with negative BC)

^c^Reported as % (No. with negative MST and negative BC/Total no. with negative MST)

^d^ Reported as % (No. with positive MST and positive BC/Total no. with positive MST)

^e^ Reported as %, (No. with positive MST and either BC or in-house PCR/No. with positive MST)

As noted, a lower cut-off value led to a substantial increase in PPV, but only a moderate decrease in sensitivity. At the cut-off value 6.0 for *S*. *aureus*, the sensitivity was 63% (12/19) and the PPV (including species-specific PCR) 65% (13/20). At the cut-off value 9.0 for *E*. *coli*, the sensitivity was 43% (16/37) and the PPV (including species-specific PCR) 86% (19/22). At the cut-off value 9.0 for *S*. *pneumoniae*, the sensitivity was 39% (5/13) and the PPV (including species-specific PCR) 86% (6/7).

When the lower MST cut-off values were used for all pathogens, we found that overall specificity and PPV clearly increased (Table 5).

**Table 5:** Sensitivities, specificities and predictive values of the Magicplex Sepsis Test (MST) at different Cq cut-off values for predicting blood culture (BC) positivity, global results for all detected pathogens.

Cq cut-off	No. of patients with positive MST	Sensitivity ^a^	Specitivity^b^	Positive predictive value^c^	Negative predictive value^d^
Any Cq value	268	8(61/128)	66(374/568)	23(61/268)	87(374/428)
< 10	211	45(58/128)	75(428/568)	27(58/211)	88(428/487)
< 9	181	42(54/128)	80(454/568)	30(55/182)	88(454/516)
< 8	127	33(42/128)	87(496/568)	34(43/128)	87(496/570)
< 6/9^e^	83	38(49/128)	96(546/568)	59(49/83)	89(546/615)

^a^Reported as % (No. with positive MST and positive BC/Total no. with positive BC)

^b^Reported as %(No. with negative MST and negative BC/Total no. with negative BC)

^c^Reported as % (No. with positive MST and positive BC/Total no. with positive MST)

^d^Reported as % (No. with negative MST and negative BC/Total no. with negative MST)

^e^ Cq cut-off 6.0 for *Staphylococcus* species and Ct cut-off 9.0 for all other species

### Cq cut-off value 6.0 for *Staphylococcus* species and 9.0 for all other pathogens

At the cut-off value 6.0 for *Staphylococcus* species and 9.0 for all other pathogens, the overall sensitivity was 38% (49/128), specificity 96% (546/568), PPV 59% (49/83), and NPV 89% (546/615).

Table 3 shows the distribution of BC and MST results after lowering the cut-off value. With the lower cut-off value, the number of MST positive results fell from 362 to 88, and the number of MST positive samples from 268 to 83. The rate of concordant positive BC in cases with a positive MST tests increased from 18% (66/362) to 59% (51/87). Among the 61 patients with concordant positive BC and MST results, 49 (80%) had a Cq value below the lower cut-off limit. The number of samples positive for *CoNS* was reduced from 138 to 13 with the lower cut-off. Polymicrobial findings were dramatically reduced, from 78 to 5 samples. All 5 were BC-positive for at least one of the pathogens detected, and in 3 both bacteria were grown in BC. Table 6 shows all cases with polymicrobial findings in BC and in MST with the lower cut-off value.

**Table 6:** Polymicrobial or disconcordant findings in blood culture and in Magicplex Sepsis Test (MST) when the Cq cut-off was set at Ct <6.0 for *staphylococci* and <9.0 for all other pathogens.

Gender, age	Blood culture result	MST result with lower cut-off	MST result according to original test result
m, 83 y	*Staphylococcus aureus*, *Klebsiella pneumoniae*	*S*. *aureus*, *Escherichia coli*	*S*. *aureus*, *E*. *coli*, *K*. *pneumoniae*
m, 86 y	*E*. *coli*, *Bacteroides fragilis*	*E*. *coli*, *B*. *fragilis*	*E*. *coli*, *B*. *fragilis*
m, 83 y	*Enterococcus faecalis*, *Enterobacter cloacae*, *Klebsiella oxytoca*	*E*. *faecalis*, *E*. *cloacae*	*E*. *faecalis*, *E*. *cloacae*
f, 69 y	*E*. *coli*, *E*. *faecalis*	*E*. *coli*	*E*. *coli*, *E*. *faecalis*
m, 68 y	*B*. *fragilis*, *Coagulase negative staphylococci (CoNS)*,	*B*. *fragilis*	*B*. *fragilis*, *CoNS*
f, 79 y	*E*.*coli*, *Proteus mirabilis*	*E*. *coli*	*E*. *coli*
m, 86 y	*E*.*faecalis*, *CoNS*	*E*. *faecalis*	*E*. *faecalis*
f, 86 y	*S*. *aureus*, *Streptococcus mitis*	negative	negative
m, 62 y	*E*.*coli*, *Clostridium perfringens*	*E*. *coli*	*E*. *coli*
m, 83 y	*CoNS*, *Propionebacterium acnes*	negative	negative
m, 39 y	*S*. *aureus*	*S*. *aureus*, *CoNS*	*S*. *aureus*, *CoNS*
m, 82 y	*E*. *coli*	*E*. *coli*, *S*. *aureus*	*E*. *coli*, *S*. *aureus*
f, 52 y	*CoNS*	*Streptococcus agalactiae*	*S*. *agalactiae*, *S*.*aureus*
M, 82 y	*Pseudomonas aeruginosa*	*S*. *aureus*	*S*. *aureus*, *CoNS*
m, 73 y	*Streptococcus pneumoniae*	*CoNS*	*CoNS*
m, 84 y	*Enterobacter sakazakii*	*K*. *pneumoniae*	*K*. *pneumoniae*

## Discussion

In this study on MST performance in a large study-cohort of patients with suspected sepsis, we found that the MST-positive rate was notably high, with a high frequency of polymicrobial results, *CoNS*, and other findings of uncertain clinical relevance. A lower Cq cut-off value significantly improved the specificity and the positive predictive value, but to the cost of a lower sensitivity.

The clinical interpretation of a positive MST with a negative BC is difficult, as there are several situations where bacterial DNA can be found in culture-negative blood. First, such a result might represent a true BSI that BC fails to detect. It is known that a positive BC is found in around only half the patients with sepsis and septic shock [9, 10]. PCR positivity, even when BC remains negative, was recently found to correlate to disease severity [26]. Second, a positive MST might be due to DNA from dead bacteria, a finding with unclear clinical significance. Third, it might represent a transient bacteremia without clinical relevance. It has been shown that bacteremia is common after dental procedures and tooth-brushing [27, 28]. Molecular pathogen detection by 16S RNA sequencing has been performed on blood at repeated intervals after dental extraction, resulting in massive findings of diverse pathogens at low concentrations [29, 30]. Fourth, it can be caused by contamination. Contamination can occur during blood sampling, resulting in positive tests for normal human skin flora such as *CoNS*. It can also occur in the laboratory; all manual work at the laboratory represents a risk for contamination, and PCR or DNA isolation reagents can be contaminated with bacterial DNA.

Based on the results of the present study, we suspect that results with positive MST and negative BC were often due to contamination or clinical irrelevant DNA-aemia. This assumption is not based on clinical evidence though, but on sepsis epidemiology. A large proportion of the MST+/BC- findings were pathogens that rarely cause community-acquired sepsis, including *CoNS* (n = 129) *Streptococcus agalactiae* (n = 26) *Stenotrophomona maltophilia* (n = 24) and *Acinetobacter baumanii* (n = 10). Since *S*.*maltophilia* and *A*. *baumanii* are not part of the normal human flora, these findings were probably due to laboratory contamination despite the use of DNA-free reagents and stringent hygiene routines. The MST method is a procedure in many steps, which might be an advantage to achieve better sensitivities, but it is also a laboratory contamination risk.

Regarding *CoNS*, most findings in the study were probably due to contamination, but not necessarily all of them. Among the samples with concordant *CoNS* positivity in MST and BC, two had growth in all four BC bottles, indicating clinical relevance. The Ct values of those samples were 4.3 resp. 6.3, which means that one of them would have been missed with the lower cut-off.

MST+/BC- results for common sepsis-causing pathogens such as *S*. *aureus*, *E*. *coli*, and *S*. *pneumoniae* could not be judged as clinically relevant or not with the present study design, as clinical data was not available.

The MST has been described in only a few previous publications, by Carrara et al [20], Loonen et al [21], and Ljungström et al [22].

Carrara and colleagues compared MST and BC results from 267 patients in the ICU, and at emergency and haematology departments. The sensitivities and specificities reported in that study were calculated on a reference standard where clinical data and other cultures were added to BC results in order to see if a positive MST represented a true BSI or not. The overall sensitivity of MST was then 65%. When compared to BC, 29 of 69 BC positive specimens were also MST positive, and consequently the sensitivity was 41%.

The study cohort in the work by Loonen et al [21] was similar to ours, i.e. patients presenting at the emergency unit with suspected sepsis. The study included 125 patients, of whom 12 were positive in both MST and BC, and a further 23, 15 of which being *CoNS*, by MST only. The sensitivity and PPV were 37% and 30%, respectively. As in our study, the low PPV resulted from the fact that many of samples were MST-positive for *CoNS*.

The study by Ljungström et al [22] was performed on 375 patients with suspected sepsis at a Swedish emergency unit. The reference standard used for calculations was a combination of BC findings, other relevant culture results, and clinical evaluation. Findings of suspected contaminants such as *CoNS* were not included in the analyses. They then reached a sensitivity of 64% and a specificity of 96%. If all findings from both MST and BC had been included in the results, the sensitivity in Ljungström’s study would have been 38%, the PPV 17% and the specificity 65%. Consequently, this study, as well as ours, shows that the MST certainly detects an important proportion of sepsis pathogens, but also a lot of bacterial DNA in blood that has doubtful clinical significance.

With the lower Cq cut-off value, i.e. 6.0 for *Staphylococcus species* and 9.0 for all other pathogens, almost all findings with uncertain clinical relevance disappeared. In contrast, the majority of MST-positive samples supported by BC findings remained positive. Recently, we showed a similar pattern for the SeptiFast test [31], where a SeptiFast test with a Cp (equivalent to Cq value) below 17.5 indicated BC positivity.

The purpose of the lower Cq cut-off value in this study was to reduce the frequency of false positive results. However, as a consequence, some true positive MST results became negative, and the sensitivity decreased. The number of BC positive samples with a concordant positive MST fell from 61/128 (48%) to 49/128 (38%) (Table 3). In addition, some MST+/BC- results that became MST-/BC- with a lower Cq cut-off, might have been clinically relevant, as we know that BC is not an optimal reference standard. Thus, we do not consider that a positive MST test with a Ct value above the lower cut-off limit should be ruled out as clinically irrelevant, but should be interpreted with caution.

A large proportion of the BC positive samples was PCR negative. MST has this problem in common with SeptiFast and other commercial PCR assays for pathogen detection in blood [14, 32–34]. One possible explanation for a negative PCR despite a positive BC might be that the sample volume of 1 mL is insufficient. The fact that BC positive samples showing a negative MST were frequently positive in only one or two bottles, whilst the majority of MST positive samples were positive in all 4 bottles, supports the fact that sample volume is an important factor. Using a larger sample volume in the MST assay would almost certainly improve sensitivity. Another explanation to some of the MST-/BC+ results is that BC positivity due to suspected contaminants such as *CoNS* are included in the analyses. Thirteen samples were BC positive for pathogens not included in the MST PCR panel, and were thus MST-/BC+.

The limited PCR panel of the assay is also a disadvantage that MST has in common with most other commercial PCR assays for pathogen detection in blood. The panel did not cover pathogens found in 10% (13/128) of the BC positive samples. In addition, there are fastidious organisms, such as *Mycobacterium tuberculosis*, *Bartonella species*, and *Legionella pneumophila*, that seldom grow in BC and that cannot be detected by MST. Another limitation of the MST is that among the more than 90 pathogens that are included in the screening step, only 27 can be identified to the species level. In our study 3% (10/372) of all pathogens detected by MST were positive only in the screening step and not further identified.

When evaluating the MST, it is relevant to compare it with the LightCycler SeptiFast test (Roche), which is, to date, the most established commercial PCR test for diagnosing BSI. In 2007–2008, our group [32] evaluated the SeptiFast test on emergency room samples with a similar study design and study population including 1,093 patients, at the same hospital. Compared to BC, the SeptiFast test showed a positive result in 9.8% (107/1,093), and sensitivities and PPV of 67% and 43% for *S*. *aureus*, 12% and 67% for *S*. *pneumoniae*, and 53% and 56% for *E*. *coli*. In the present study, MST with the lower Ct cut-off value showed a positive result in 12% (83/696) and sensitivities and PPV of 63% and 60% for *S*. *aureus*, 39% and 71% for *S*. *pneumoniae*, and 43% and 73% for *E*. *coli*. Consequently, the MST test with the lower Cq cut-off value showed at least as good performance as the SeptiFast test, and was clearly superior to the SeptiFast test for detection of *S*. *pneumoniae* BSI.

Other commercially available molecular assays include SepsiTest (Molzym) and VYOO (Analytik Jena), with reported sensitivities of 37%-87% [15, 35] for SepsiTest and 38%-60% [14, 36] for VYOO. Thus, these assays and MST appear to have similar sensitivities. However, comparison between studies is difficult due to factors such as differences in the clinical condition of the patients, the number of patients receiving antibiotic treatment before taking the blood samples, the number of blood cultures taken, and whether or not findings of *CoNS* and other suspected contaminants in BC were included. The specificities are even more difficult to compare between studies. In some studies, all suspected contaminants have been excluded before performing analyses, and in many studies the reference standard used for the calculations has included more data than just BC positivity.

The latest commercial molecular assay, the IRIDICA PCR/electrospray ionization—mass spectrometry assay (Abbott), has shown promising sensitivity in BSI pathogen detection; 73%-81% [18, 19]. The method uses a large blood sample volume, 5 mL, which is probably the main reason for its increased sensitivity. This method uses mass-spectrometry that allows a broad-range detection, which is an advantage compared to methods, such as MST, with limited PCR panels. The specificity of the IRIDICA test is unclear, as it has been found to detect many pathogens that have not been supported by BC, in many cases with unknown significance [18, 19].

The present study has some strengths and limitations. The major strength is that the MST was run on consecutive patients in a large, defined study population.

The main limitation is that we did not have access to clinical data other than gender, age and BC results for the study cohort, according to the decision of the Regional Medical Ethics Review Board. However, in order to improve the relevance of this evaluation, we included species-specific PCR for the most frequently detected pathogens. It was our assumption that if the pathogen was detected by two different PCR methods, it was likely to be a relevant finding.

## Conclusions

In conclusion, our study showed that the Cq value can be used as a tool in the interpretation of a positive MST result, but we do not suggest a strict application of the lower cut-off, as it would affect the sensitivity. However, even with the lower cut-off the sensitivity of the MST was comparable with other commercial molecular methods in the same field of application. Similar diagnostic strategies, taking quantitative DNA data into account, could probably be used in the interpretation of other molecular methods as well.

Future studies on commercial tests for microbiological sepsis diagnosis, should further establish the role and clinical importance of DNA quantification.

## Supporting Information

S1 Fig Supporting data file containing all raw data used in the statistical analyses. (XLSX)
